# PoDFEd: Podiatrists and Diabetes Footcare Education Survey - How do Australian podiatrists provide diabetes education?

**DOI:** 10.1186/s13047-020-0376-4

**Published:** 2020-02-18

**Authors:** Julia Yuncken, Terrance Haines, Renerus J. Stolwyk, Cylie M. Williams

**Affiliations:** 1 Monash University Department of School of Primary and Allied Health Care, McMahon’s Rd, Melbourne, VIC 3199 Australia; 2 School of Psychological Sciences, Monash University., Melbourne, Australia; 3 Monash Institute of Cognitive and Clinical Neurosciences, Melbourne, Australia; 4 Monash-Epworth Rehabilitation Research Centre, Melbourne, Australia; 5 Peninsula Health, Allied Health, Melbourne, VIC 3199 Australia

**Keywords:** Podiatrists, Diabetes, Education, Complications, Foot

## Abstract

**Background:**

Podiatrists provide care and education to people with diabetes. This often includes the use of education relating to complications of the disease and how to prevent them. It is currently unknown how Australian podiatrists provide this education. This study aimed to describe the foot related diabetes education being delivered to people with diabetes within the Australian podiatry setting.

**Methods:**

This cross-sectional cohort study contacted Australian podiatrists to complete an online survey regarding their provision of diabetes education. The Qualtrics online survey application was advertised to Australian podiatrists via social media, at state conferences and through the Australian Podiatry Association and other similar association group emails. A multivariate stepwise progression was utilised to collate and decipher data. A chi-squared test was used to determine significant links between podiatrist’s method of education, demographic variables and topics of education.

**Results:**

Findings linked the use of visual, written, generic handout and individualised handouts to various components of education and demographic information of Australian podiatrists. Verbal education had no significant links to demographic and topics of education relating to diabetes.

**Conclusions:**

This paper discovered a range of topics covered and methods used by Australian podiatrists during consultations with patients with diabetes.

## Introduction

Diabetes is a complex disease affecting 425 million worldwid e[1]. In Australia, estimates suggest that 5-6% of the population has diabetes, on which the public health system spends (USD)$5650 spent per person per year [1]. Complications from diabetes include cardiovascular disease, kidney disease, vision loss, and foot ulceration and amputation [2]. A mainstay of diabetes treatment is diabetes related education. Several key diabetes groups, such as the International Working Group on the Diabetic Foot, advocate the use of regular culturally appropriate education for all people with diabetes [1–4]. The aim of ongoing education is to reduce the possible complications of the disease for both the individual and the health care system [3, 5].

Podiatrists commonly provide education throughout consultations as standard practice. Education topics may include complications of the disease affecting the lower limb such as vascular disease, neuropathic changes, wound care, footwear assessment and general foot care. They may tailor this education according to a person’s health, ability or complications relating to their diabetes. The education provided by health practitioners should engage the person to incorporate diabetes self-care strategies to decrease complications of the disease [6].

Behavioural change via education is possible [7]; however, the current educative practices of practitioners are unknown. Education methods may vary across different podiatry workplace settings. The methods may include videos, written information that is generic or tailored to each person, verbal discussion, or a combination of each. There is some evidence to suggest that some methods of education are more valuable than others within the context of diabetes education [8]. Currently, however, within the Australian podiatry setting, it is unknown what education topics and methods of education delivery are provided to people with diabetes. It is also unknown if there are barriers to the provision of diabetes related education, from the podiatrist’s perspective.

The primary aim of this research was to describe foot-related diabetes education being delivered to people with diabetes within the Australian podiatry setting. The secondary aims were to describe how podiatrists provide education and identify factors associated with how podiatrists provide diabetes related foot education content and methods.

## Method

### Study design

This research was a cross-sectional design study. The Monash University Human Ethics Committee approved this research (HREC: 12777). The CHERRIES (Checklist for Reporting Results of Internet E-Surveys) was utilised in reporting the survey outcomes [9].

### Participants and setting

All podiatrists (*n* = 5051) working within Australia between of 18th April-17th of August, 2018, were eligible to take part [10]. Advertising of the survey took place at local Australian state conferences, via social media (Facebook, Twitter, LinkedIn) and via email flyers to podiatry services and clinics. Further advertising was undertaken via the Australian Podiatry Association, Advanced Practising Podiatrists – High Risk Foot Group, Diabetic Foot Australia newsletters and websites. There was an incentive of a $20(AUD) voucher for 10 participants that was drawn at the closure of the survey. Names for prize draw were collected in a way that was not linked with their answers.

### Measurements

A single-round online (Qualtrics) [11] questionnaire was developed to collect information on how podiatrists provide foot related diabetes education within the clinical setting (Supplementary File 1). There were two components to data collection: the podiatrist’s demographic data and education-related data. No identifying data were collected. Participants were informed of the Qualtrics data storage and privacy policies. These questions were displayed over six screens with screen breaks between question groupings or where transitions between open and closed questions were used. Podiatrist data focused on years of experience, work setting, age, gender and percentage of their clinical load directly linked with people who have diabetes. Education related data focused on method and topic of education provided, barriers to providing education and self-perceived outcomes of diabetes education. Initially, podiatrists were asked open ended questions in a non-prompted manner regarding the topics and methods used. In any non-prompted questions, the participant was not given a choice of answers to minimise the impact this might have on responses recorded (e.g. Question 10). In prompted questions, the participant was provided with a set of response categories to choose from (e.g. Question 11). The survey was conducted in this manner to gain representative answers about what occurs in current practice. Trialling of the survey on three podiatrists was conducted for content and language checking before advertising.

### Procedure

Distribution and advertising of the survey to Australian podiatrists occurred over five months during 2018. Each podiatrist consented to take part and complete the survey online; completion was anonymous.

The responses were collected using Qualtrics online survey software. Participants were able to withdraw from the survey at any point by closing the browser. Results from respondents who withdrew mid-survey were treated as missing data for any remaining non-completed variables. Internal fidelity was promoted by not allowing the respondent to skip to the next section without completing the prior section. No additional fields were used to understand unique completions, cookies were used to allow participants to return back to their response within 4 h of partial completion only. No IP checking was used.

### Analysis

Data were analysed using Stata 13 [12]. Descriptive statistics were used to describe each variable. Open and closed ended questions were compared using a Chi-Square comparison. Factors associated with education method were explored using forward stepwise multivariable regression. Factors examined related to when, where, or by whom these methods were being used, along with other methods concurrently being used, number of topics discussed, topics of discussion, and podiatrist perception that the person was retaining information. Variables considered for introduction to the multivariable model were those with univariate associations where *p* < 0.2 [13]. The multivariable model was developed by introducing variables one at a time based on the variable with the lowest *p*-value. This forward step introduction continued, and the Akaike information criterion (AIC) was used at each step to estimate the relative quality of successive models [12]. Where the AIC increased, the variable was removed, and the next variable was introduced. The variables continued to be introduced where the AIC continued to lower and were removed if the AIC elevated, and all variables identified were trialled within the model [13]. Complete case analysis was applied, and only data from respondents where all treatment options and demographic data were answered were included.

Open and closed ended questions about the topics of education covered and method used were firstly coded into themes similar to those asking respondents to nominate a response. These were quantified and compared using a Chi-Square statistic with Stata [12]. Open ended question responses about the topics and methods of education were initially grouped based on similar response. These questions were grouped by a single researcher (JY) and where there was ambiguity, these were planned for checking with an additional researcher (CMW) however there were no ambiguous answers. These themes were quantitatively described.

## Results

There were 512 (10% of 5051 Australian podiatrists) responses with full or partial survey completion [10]. Podiatrists working in Victoria were the largest group of responders (*n* = 194, 38% of 512). The majority of the respondents were female (*n* = 327, 64% of 512). Table 1 reports the demographic data. Greater than half of all respondents (*n* = 227, 44% of 512) reported that greater than 50% of their caseload included people with diabetes, and 54% (*n* = 277) of respondents worked in private practice (Table 1).

**Table 1:** Demographics of participants – n (%)

	Total responses *n*=512	ACT *n*=8, 1% of total responses	NSW *n*=104, 20% of total responses	NT *n*=5, 1% of total responses	QLD *n*=91, 18% of total responses	SA *N*=35, 7% of total responses	TAS *n*=15, 3% of total responses	VIC *n*=194, 38% of total responses	WA *N*=60, 12% of total responses
***Gender***	n(%)	n(%)	n(%)	n(%)	n(%)	n(%)	n(%)	n(%)	n(%)
Male	181 (35%)	1 (12%)	53 (51%)	1 (20%)	29 (32%)	12 (35%)	3 (20%)	59 (30%)	23 (38%)
Female	329 (64%)	6 (76%)	51 (49%)	4 (80%)	62 (68%)	23 (65%)	12 (80%)	134 (69%)	37 (62%)
Prefer not to answer	2 (1%)	1 (12%)	0 (0%)	0 (0%)	0 (0%)	0 (0%)	0 (0%)	1 (1%)	0 (0%)
***Age group***	n(%)	n(%)	n(%)	n(%)	n(%)	n(%)	n(%)	n(%)	n(%)
Under 25	61 (12%)	0 (0%)	16 (15%)	2 (40%)	4 (4%)	1 (2%)	4 (27%)	28 (15%)	6 (10%)
25 - 35	235 (46%)	4 (50%)	47 (45%)	0 (0%)	40 (44%)	14 (40%)	8 (53%)	100 (52%)	22 (37%)
36 – 45	109 (21%)	2 (26%)	25 (24%)	1 (20%)	22 (24%)	9 (26%)	1 (7%)	32 (16%)	17 (28%)
46 - 55	81 (16%)	1 (12%)	10 (9%)	0 (0%)	20 (22%)	9 (26%)	2 (13%)	26 (13%)	13 (22%)
56 +	26 (5%)	1 (12%)	6 (7%)	2 (40%)	5 (6%)	2 (6%)	0 (0%)	8 (4%)	2 (3%)
***Working environment***	n(%)	n(%)	n(%)	n(%)	n(%)	n(%)	n(%)	n(%)	n(%)
Acute Setting	83 (16%)	1 (12%)	12 (12%)	2 (40%)	20 (22%)	9 (26%)	0 (0%)	31 (15%)	8 (13%)
Sub-acute Setting	24 (5%)	0 (0%)	1 (1%)	0 (0%)	7 (7%)	3 (9%)	3 (20%)	7 (4%)	3 (5%)
Community Health	104 (20%)	5 (63%)	6 (5%)	0 (0%)	18 (20%)	0 (0%)	3 (20%)	67 (35%)	5 (8%)
Private Practice	280 (55%)	2 (25%)	81 (77%)	1 (20%)	40 (44%)	20 (56%)	9 (60%)	83 (43%)	44 (74%)
Other*	21 (4%)	0 (0.00%)	4 (4%)	2 (40%)	6 (7%)	3 (9%)	0 (0%)	6 (3%)	0 (0%)
***Podiatry Qualification***	n(%)	n(%)	n(%)	n(%)	n(%)	n(%)	n(%)	n(%)	n(%)
Diploma	53 (10%)	1 (13%)	12 (12%)	1 (20%)	17 (19%)	7 (20%)	1 (7%)	6 (3%)	8 (13%)
Bachelor	362 (71%)	6 (75%)	74 (71%)	2 (40%)	68 (75%)	27 (78%)	10 (67%)	134 (69%)	41 (68%)
Masters	77 (15%)	0 (0%)	14 (13%)	1 (20%)	3 (3%)	0 (0.00%)	2 (13%)	50 (26%)	7 (12%)
Other*	20 (4%)	1 (12%)	4 (4%)	1 (20%)	3 (3%)	1 (2%)	2 (13%)	4 (2%)	4 (7%)
	*n*=510	*n*=8	*n*=104	*n*=5	*n*=91	*n*=35	*n*=15	*n*=192	*n*=60
***Years of practice***	n(%)	n(%)	n(%)	n(%)	n(%)	n(%)	n(%)	n(%)	n(%)
0-2	71 (14%)	0 (0%)	18 (17%)	2 (40%)	8 (9%)	3 (9%)	4 (27%)	29 (15%)	7 (12%)
3-5	80 (16%)	1 (13%)	27 (26%)	0 (0%)	9 (10%)	4 (11%)	1 (6%)	28 (14%)	10 (16%)
6-10	133 (26%)	3 (37%)	23 (22%)	0 (0%)	29 (32%)	6 (17%)	4 (27%)	59 (31%)	9 (15%)
11-15	77 (15%)	2 (25%)	8 (8%)	0 (0%)	12 (13%)	8 (23%)	4 (27%)	37 (20%)	6 (10%)
15+	149 (29%)	2 (25%)	28 (27%)	3 (60%)	33 (36%)	14 (40%)	2 (13%)	39 (20%)	28 (47%)
	*n*=488	*n*=8	*n*=104	*n*=5	*n*=85	*n*=33	*n*=14	*n*=184	*n*=55
***% of patients with diabetes (per week)***	n(%)	n(%)	n(%)	n(%)	n (%)	n(%)	n(%)	n(%)	n(%)
< 25%	91 (19%)	0 (0%)	21 (21%)	0 (0%)	15 (18%)	11 (33%)	2 (13%)	30 (16%)	12 (22%)
26-50%	164 (34%)	3 (37%)	42 (41%)	1 (20%)	22 (26%)	8 (24%)	5 (38%)	63 (34%)	20 (36%)
51-75%	116 (24%)	3 (37%)	24 (23%)	2 (40%)	20 (23%)	6 (19%)	3 (21%)	45 (25%)	13 (24%)
76%+	117 (23%)	2 (26%)	17 (15%)	2 (40%)	28 (33%)	8 (24%)	4 (38%)	46 (25%)	10 (18%)

*Other included: Aged Care facility, Non-Governmental Organisation, Remote Communities, University, Aboriginal Medical Services, Surgeon

Respondents reported preferring the grouped themes of verbal education (*n* = 426, 94% of 477) and verbal education combined with written reinforcement education (*n* = 211, 47% of 477) when firstly describing preferred education methods in the non-prompted, open ended questions. When completing the prompted, closed answer questions, respondents provided further detail on education methods and reported more frequent use of other additional education methods as described in Table 2. There was a significant difference between the non-prompted and promoted question responses by the podiatrist (Tables 2), particularly for verbal, written, and handout – proforma/brochure methods (*p* < 0.01).

**Table 2:** Difference in response frequency between unprompted, open ended questions and prompted questions for methods of diabetes education delivery (Questions 10 & 11)

Method of education	Coded responses to unprompted, open ended questions n(%) of 477	Responses to prompted questions n (%) of 451	X^2^,(df), p
Written	0 (0%)	211 (47%)	286.18 (1), < 0.01
Verbal	217 (46%)	426 (94%)	258.89 (1), < 0.01
Visual aid/video	0 (0%)	61 (14%)	65.81 (1), < 0.01
Handout - Individualized	0 (0%)	99 (< 1%)	114.92 (1), < 0.01
Handout - Proforma/Brochure	0 (0%)	232 (51%)	324.42 (1), < 0.01
Verbal + Written	210 (44%)	0 (0%)	254.12 (1), < 0.01
Verbal + Written + Visual	16 (< 1%)	0 (0%)	13.48 (1), < 0.01
Verbal + Visual	6 (< 1%)	0 (0%)	3.92 (1), < 0.01
No Education	1 (< 1%)	0 (0%)	0.95 (1), 0.33
Other (please specify)	28 (< 1%)	22 (< 1%)	0.27 (1), 0.60

Respondents indicated in closed ended questions that they routinely educated on topics including neuropathy (*n* = 406, 98% of 413), vascular (*n* = 396, 96% of 413), footwear (n = 396, 96% of 413), general foot health (*n* = 370, 90% of 413), and ulceration risks (*n* = 386, 93% of 413). Within the open ended questions these topics were much more evenly reported by podiatrists as discussed in their consultation - neuropathy (*n* = 217, 52% of 420), vascular (*n* = 211, 50% of 420), footwear (*n* = 98, 23% of 420), general foot health (*n* = 193, 46% of 420), and ulceration risks (n = 98, 2% of 420). Reporting found multiple other topics in the open ended questions, such as discussion of medications, Blood Glucose Level (BGL) management and referrals to other health care professionals (Table 3). Results indicating podiatrists commonly covering several topics during the consultation were high, with most podiatrists covering two or more topics per consult (*n* = 277, 89%).

**Table 3:** Difference in response frequency between unprompted, open ended questions and prompted questions for topics of diabetes education (Questions 13 & 14)

Topic of education	Coded responses to unprompted, open ended question n(%) of 420	Responses to prompted questions n (%) of 413	X^2^ (df), *P* Values
Vascular	211 (50%)	396 (96%)	0.0 (1), 0.99
Neuropathy	217 (52%)	406 (98%)	0.10 (1), 0.75
Ulceration risks	98 (23%)	386 (93%)	417.82 (1), < 0.01
Footwear	98 (23%)	396 (96%)	451.15 (1), < 0.01
General skin and nail care	193 (46%)	370 (90%)	179.00(1), < 0.01
Blood sugar levels	96 (2%)	359 (87%)	342.26 (1), < 0.01
Physical activity	22 (< 1%)	303 (73%)	403.34 (1), < 0.01
Smoking	8 (< 1%)	268 (65%)	370.05 (1), < 0.01
Dietary	17 (< 1%)	232 (56%)	267.51 (1), < 0.01
Medication	1 (< 1%)	0 (0%)	0.99 (1), 0.32
Referrals	7 (< 1%)	0 (0%)	5.09 (1), 0.02
Other (please specify)	15 (< 1%)	17 (< 1%)	0.05 (1), 0.82

Individualised handout use was associated with less use of other written information (OR = 0.05, 95%CI = 0.02 to 0.15, *p* < 0.001), less use of visual media (OR = 0.02, 95%CI = 0.00 to 0.09, *p* < 0.001), and increased number of methods used (OR = 40.85, 95%CI = 15.89, 104.99, *p* < 0.001) (Table 4). Written education use was associated with greater number of methods used (OR = 54.88, 95%CI = 28.58, 105.37, *p* < 0.001), less use of individualised handouts (OR = 0.03, 95%CI = 0.01, 0.09, *p* < 0.001), and less use of generic handouts (OR = 0.02, 95%CI = 0.01, 0.05, p < 0.001) (Table 5). Visual media use was associated with greater number of methods used (OR = 9.32, 95%CI = 5.15, 16.88, p < 0.001), greater use of group and individual education combined (OR = 2.37, 95%CI = 1.08, 5.2, *p* = 0.03), and less use of individualised handouts (OR = 0.11, 95%CI = 0.04, 0.31, p = < 0.001) (Table 6). There were no factors associated with provision of verbal education (Table 7). Generic handout use was associated with greater number of methods used (OR = 497.83, 95%CI = 42.02, 5898.41, *p* < 0.001), less with the use of written education (OR = 0.001, 95%CI = 0.00, 0.2, p < 0.001), and less with the use of verbal education (OR = 0.01, 95%CI = 0.00, 0.23, p < 0.001). (Table 8).

**Table 4:** Variables associated with individual handout use

Handouts	Odds Ratio [95% CI]	*p* value
Use of written information (yes/no)	**0.05, [0.02 0.15]**	***P*< 0.001**
Use of visual media information (yes/no)	**0.02, [0.00, 0.09]**	***P*< 0.001**
Number of methods	**40.85, [15.89, 104.99]**	***P*< 0.001**
Education provided in a group setting (yes/no)	14.61, [0.42, 503.24]	0.19
Smoking education provided (yes/no)	1.23, [0.60, 2.52]	0.56
Skin education provided (yes/no)	0.30, [0.10, 0.91]	0.33
Footwear education provided (yes/no)	11.51, [0.32, 410.23]	0.18
Podiatrist perceived education was retained (yes/no)	1.42, [0.71, 2.85]	0.33

**Table 5:** Variables associated with written education

Written	Odds Ratio [95% CI]	*p* value
Number of methods	**54.88, [28.58, 105.37]**	***p*< 0.001**
Total topics of education provided	0.88, [0.67, 1.16]	0.37
Use of individualised handouts (yes/no)	**0.03, [0.01, 0.09]**	***p*< 0.001**
Use of generic handouts (yes/no)	**0.02, [0.01, 0.05]**	***p*< 0.001**
Ulcer education used (yes/no)	2.02, [0.48, 8.44]	0.33
Skin education used (yes/no)	4.61, [1.4, 15.21]	0.10
Blood Sugar Level education used (yes/no)	1.32, [0.41, 4.22]	0.65
Age of podiatrist	0.82, [0.59, 1.14]	0.24

**Table 6:** Variables associated with visual media use

Visual	Odds Ratio [95% CI]	*p* value
Number of methods used	**9.32, [5.15, 16.88]**	***p*< 0.001**
Both group and individual education provided	**2.37, [1.08, 5.2]**	**0.03**
Neither group or individual education provided	0.15, [0.02, 1.38]	0.09
Use of individualised handouts (yes/no)	**0.11, [0.04, 0.31]**	***p*< 0.001**
Smoking education used (yes/no)	0.88, [0.38, 2.01]	0.76

**Table 7:** Variables associated with verbal education

Verbal	Odds Ratio [95% CI]	*p* value
Number of methods used	5.24, [0.91, 30.34]	0.06
Total topics used	1.04, [0.73, 1.48]	0.83
Practitioner working in private practice setting	0.24, [0.02, 2.6]	0.24
Practitioner working in community health setting	0.27, [0.02, 3.43]	0.31
Use of individualised handouts (yes/no)	2.67, [0.42, 16.91]	0.30
Use of generic handouts (yes/no)	0.25, [0.02, 2.77]	0.26

**Table 8:** Variables associated with generic handout use

Generic Handouts	Odds Ratio [95% CI]	*p* value
Number of methods used	**497.83, [42.02, 5898.41]**	***p*** **< 0.001**
Total topics used	1.1, [0.63, 0.75]	1.60
Practitioner working in private practice setting	0.26, [0.03, 1.99]	0.20
Practitioner working in community health setting	2.48, [0.4, 15.6]	0.33
Use of written education (yes/no)	**0.001, [0.00, 0.2]**	***p*< 0.001**
Use of verbal education (yes/no)	**0.01, [0.00, 0.23]**	***p*** **< 0.004**
Podiatrist percentage of caseload that is diabetes related	0.42, [0.17, 1.04]	0.06
Podiatrist perceived education was retained (yes/no)	0.33, [0.09, 1.27]	0.11

## Discussion

Australian podiatrists who participated in this research provided diabetes education with varied methods. Many of these methods have mixed evidence for their use [14]. Further to this, there is a mixed understanding of how the relevant methods translate to behavioural change [15]. This exploration provided an insight into current professional practice, thereby enabling understanding of what behaviours may be challenged or expanded, in light of new educational or behavioural strategies to prevent diabetes complications. Australian podiatrists are currently using a variety of educational methods about the complexities of diabetes related foot disease and prevention strategies (questions 10 & 11) [4]. During education, there also appeared that many podiatrists cover numerous education topics relating to diabetes related foot disease (questions 13 & 14). Some methods of education also appeared impacted by age, workplace setting and variation in education methods or topics as provided by the podiatrist.

The podiatrists who participated in this research covered a variety of topics of education such as vascular and neuropathic complications of diabetes, lifestyle factors such as smoking cessation, encouragement of self-foot care, encouragement of dietary changes, and diabetes specific topics of BGL monitoring. These podiatrists also reported covering multiple topics of education per consult to this particular group of people attending podiatry consultations. The use of multiple topics during a consultation may be confusing the main education message. This confusion, in turn, may be diluting the predominant message the podiatrist wishes to provide based on the perceived clinical need [16]. Multiple studies highlight the use of simple, culturally sensitive education relating to diabetes and its benefits [17]. The International Working Group on the Diabetic Foot provides easily accessible instructions to clinicians supporting the use of culturally diverse and structured education to this particular group of people attending podiatry consultations [4]. Nevertheless, there is no known volume of information that best provides the knowledge needed for behaviour change without overwhelming a person who has diabetes.

This research also explored the associations of different methods of education. Podiatrists who responded commonly used individualised handouts, which were associated with the use of both written and visual media methods of education, while the written education was also associated with using generic handouts. The use of differing styles of education may be beneficial, as it is well known that adults have different learning styles [18]. Chronic disease management and education have benefited from the use of differing educational method such as verbal, written and visual methods [18]. The use of mixed methods of education has some evidence supporting diabetes related foot outcomes, including ulcer management, amputation and prevention strategies [19]. In the context of diabetes, current literature suggests that education and engagement strategies based on the Health Belief Model, or the use of motivational interviewing techniques have a more significant behavioural change impact [18]. These engagement and motivational strategies aim to ensure people fully understand the impact of their persistent disease state and enable them to engage in self-motivational strategies to make changes that have a positive impact on their life. Future research in this area should be structured in a way to capture these contemporary methods of engagement to prevent diabetes related foot disease.

There were no significant associations found between the education methods and the podiatrists' caseload. The lack of significant findings may be a reflection of the preferred education method during any presentation. It is also unclear if preferred education methods are a reflection of the differing levels of foot health risk exposure from the podiatrist during their typical duties. This present study did not consider nor collect any data on the severity of foot problems commonly treated. The varying levels of foot health risk exposure may be a factor for future researchers to consider. In conjunction to links between settings and locations of the practitioner and if this impacts on the severity of risk and foot complications.

The primary limitation of this study is relating to the number of respondents. There was 512 (10% of 5051) responses to the survey, with New South Wales and Victoria having the highest response rates (n=103, 7% and n = 193, 11% respectively). These are the two Australian states that have the highest percentage of registered podiatrists at the time of the survey. There is potential that the methods used in these states may be the results of a similar education or may skew the results in a way that is not representative of methods used in other states. Feedback from participants highlighted the broad scope of practice that podiatrists can enjoy, with some reporting they rarely treated those with diabetes. There also appeared to be some confusion around the wording of questions relating to the education topics provided in consultations. Some participants remarked “I just answered this question” in relation to the use of open and closed questions such as questions 10 &11. This confusion may have skewed results creating biased outcomes when the intended aim of the question was possibly misunderstood. With further testing of the survey prior to requesting participants to complete it, this may have been further limited. Despite this survey questioning only the targeted population, it is not possible to generalise these results to all Australian podiatrists due to the limited sample size [20]. The ability to generalise results will also depend on bias within the study; for example, the higher response rate from podiatrists in Victoria and New South Wales [20]. Despite this, the associations between how podiatrists responded and the education provided may still be described. Studies, including a greater number of podiatrist responses and including a more considerable variation in workplace settings, may impact on the results. Further research should consider adopting an approach which enables data collection across different sectors, settings and countries.

It remains unknown how different approaches to providing education impact the long-term outcomes for people with diabetic foot. This research did not attempt to explore how education relates to behaviour change within this cohort or the cohort of people who attend podiatry services. The use of techniques such as Motivational Interviewing were not reported within this study, however it has been shown to have some positive effects on retention of information and change in health behaviours [21]. In the absence of known education strategies that do have an impact on foot health, podiatrists should not take these findings as a reason to abandon education practices. There is evidence supporting the notion that education provision at the right time and in the right setting is crucial to behaviour change [22]. Podiatrists will not know if their consultation will be this tipping point where a person will choose to make positive change.

## Conclusion

This research highlights the depth of topics and methods used by Australian podiatrist concerning diabetes related foot disease. Podiatrists provide education in a variety of means, however may consider broadening their methods in light of limited education impact or taking into account the education preference and needs of the person who has disease.

## Supplementary information

**Additional file 1.** Supplementary File 1. Survey Questions.

## Data Availability

Data and materials were collated and maintained confidentially.

## Abbreviations

AUD: Australian dollar
BGL: Blood Glucose Level
CHERRIES: Checklist for Reporting Results of Internet E-Surveys
USD: United States of America dollar
