# Supplementary material for: PoDFEd: Podiatrists and Diabetes Footcare Education Survey - How do Australian podiatrists provide diabetes education?
Source: J Foot Ankle Res. 2020 Feb 18;13:8. doi: 10.1186/s13047-020-0376-4 (PMC7029509; doi:10.1186/s13047-020-0376-4)
Supplement: Supplementary file 1 — Additional file 1. Supplementary File 1. Survey Questions. [file 13047_2020_376_MOESM1_ESM.docx]

Supplementary File 1. Survey Questions

Demographics:

1. Age

(<25) (26-35) (36-45) (46-55) (55-65) (65+)

2. Years Practiced

(0-2) (3-5) (6-10) (11-15) (>15)

3. Entry level qualifications to work as a podiatrist

Podiatry - Diploma, Bachelor, Masters

Other (please comment)

4. Gender

Male/Female/decline to answer

5. State

ACT, NSW, NT, QLD, SA, TAS, VIC, WA

6. Work Place

Metro/Regional/Rural

Acute/Sub-Acute/Community Health/Private Practice

7. How many hours per week do you work as a clinical podiatrist?

(0-10) (11-20) (21-30) (31-40) (40+)

Work:

8. What % caseload of people with diabetes do you see per week?

(<25%) (26-50%) (51-75%) (76%+)

9. What % caseload of ulcerated/amputee diabetes patients do you see per week?

0-10%, 11-20%, 21-30%, 31-40%, 41-50%, 51-60%, 61-70%, 71-80%, 81-90% 91-100%

Diabetes Education:

10. What type of education do you typically provide? Eg handouts (open ended)?

11. What % of mode of diabetes education do you provide for your patients with diabetes?

Written

Verbal

Visual

Handout – Individualised

Handout - Proforma

12. Do you provide Group or Individual education?

Group/Individual/Both/Neither

13. What diabetes related educational content do you provide during your consultations? Eg vascular risks, neurological changes. (open ended)

14. What % of content of education do you provide?

Vascular

Neuropathy

Ulceration

Footwear

General Care

BSL

Physical Education

Smoking

Dietary

Charcot

15. Do you feel patients have difficulty retaining the diabetes education you provide?

16. What do you feel are the barriers to providing diabetes education? (open ended)

17. What do you feel would aid in providing better diabetes education?

More time

Better resources

Greater practitioner skill

Other (please comment)

18. Do you upskill your diabetes education knowledge?

Yes/No

18a. If Yes: How?

Conference

Webinars

Journal articles

Other (please comment)

18b. If no: What are the barriers to practitioner upskilling?

Time commitment

Cost

Don’t know where to source

Other (please comment)
